# Largest Amplitude of Glycemic Excursion Calculating from Self-Monitoring Blood Glucose Predicted the Episodes of Nocturnal Asymptomatic Hypoglycemia Detecting by Continuous Glucose Monitoring in Outpatients with Type 2 Diabetes

**DOI:** 10.3389/fendo.2022.858912

**Published:** 2022-04-14

**Authors:** Shoubi Wang, Zhenhua Tan, Ting Wu, Qingbao Shen, Peiying Huang, Liying Wang, Wei Liu, Haiqu Song, Mingzhu Lin, Xiulin Shi, Xuejun Li

**Affiliations:** ^1^ Department of Endocrinology and Diabetes, Xiamen Diabetes Institute, Xiamen Clinical Medical Center for Endocrine and Metabolic Diseases, Xiamen Diabetes Prevention and Treatment Center, Fujian Key Laboratory of Diabetes Translational Medicine, The First Affiliated Hospital of Xiamen University, School of Medicine, Xiamen University, Xiamen, China; ^2^ Fujian Provincial Key Laboratory of Ophthalmology and Visual Science, Eye Institute of Xiamen University, School of Medicine, Xiamen University, Xiamen, China; ^3^ Xiahe Branch of the Zhongshan Hospital Affiliated to Xiamen University, Xiamen, China; ^4^ The School of Clinical of Medicine, Fujian Medical University, Fuzhou, China

**Keywords:** nocturnal asymptomatic hypoglycemia, largest amplitude of glycemic excursion, self-monitoring blood glucose, continuous glucose monitoring, outpatients with type 2 diabetes

## Abstract

**Aims:**

Nocturnal asymptomatic hypoglycemia (NAH) is a serious complication of diabetes, but it is difficult to be detected clinically. This study was conducted to determine the largest amplitude of glycemic excursion (LAGE) to predict the episodes of NAH in outpatients with type 2 diabetes.

**Methods:**

Data were obtained from 313 outpatients with type 2 diabetes. All subjects received continuous glucose monitoring (CGM) for consecutive 72 hours. The episodes of NAH and glycemic variability indices (glucose standard deviation [SD], mean amplitude of plasma glucose excursion [MAGE], mean blood glucose [MBG]) were accessed *via* CGM. LAGE was calculated from self-monitoring blood glucose (SMBG).

**Results:**

A total of 76 people (24.3%) had NAH. Compared to patients without NAH, patients with NAH showed higher levels of glucose SD (2.4 ± 0.9 mmol/L vs 1.7 ± 0.9 mmol/L, p <0.001), MAGE (5.2 ± 2.1 mmol/L vs 3.7 ± 2.0, p<0.001) and LAGE (4.6 ± 2.3 mmol/L vs 3.8 ± 1.9 mmol/L, p=0.007), and lower level of MBG (7.5 ± 1.5 mmol/L vs 8.4 ± 2.2 mmol/L, p=0.002). LAGE was significantly associated with the incidence of NAH and time below rang (TBR) in model 1 [NAH: 1.189 (1.027-1.378), p=0.021; TBR: 0.008 (0.002-0.014), p=0.013] with adjustment for age, BMI, sex, work, hyperlipidemia, complication and medication, and in model 2 [NAH: 1.177 (1.013-1.367), p=0.033; TBR: 0.008 (0.002-0.014), p=0.012] after adjusting for diabetes duration based on model 1, as well as in model 3 [NAH: 1.244 (1.057-1.464), p=0.009; TBR: 0.009 (0.002-0.016), p=0.007] with further adjustment for HbA1c based on model 2. In addition, no significant interactions were found between LAGE and sex, age, HbA1c, duration of diabetes, BMI and insulin therapy on the risk of NAH. The receiver operator characteristic (ROC) curve shows the ideal cutoff value of LAGE for the prediction of NAH was 3.48 mmol/L with 66.7% sensitivity, 50% specificity and 0.587 (95% CI: 0.509-0.665) of area under the ROC curve.

**Conclusions:**

High glycemic variability is strongly associated with the risk of NAH. The LAGE based on SMBG could be an independent predictor of NAH for outpatients with type 2 diabetes, and LAGE greater than 3.48 mmol/L could act as a warning alarm for high risk of NAH in daily life.

## Introduction

Hypoglycemia is a serious complication of diabetes mellitus, which could contribute to “dead in bed” syndrome, neurological damage (poorer cognitive function, spatial memory dysfunction, neuron damage, and epilepsy), and psychological impact (negative psychosocial consequences, undesirable compensatory behaviors, unforeseen anxiety, and poor sleep) (1). With the implementation of intensive glucose control over the years, the morbidity of hypoglycemia is relatively higher (2, 3). Accordingly, high rates of different degrees of hypoglycemia episodes, namely severe events [1.0~16.9% (4–8)], moderate severity events [17~46% (4–7)] and mild events [46~58% (5–7)] have been reported. Simultaneously, hypoglycemia was assigned as the cause of death in 4% (9), 7% (10), and 10% (11) in population-based registers. As almost 50% of all severe hypoglycemia episodes occur at nighttime during sleep with unawareness, nocturnal asymptomatic hypoglycemia (NAH) has been especially emphasized (12). Recurrent episodes of asymptomatic hypoglycemia can increase the risk of severe hypoglycemic episodes (13), contributing to life-threatening events, such as major macrovascular events, major microvascular events, death from cardiovascular disease, and death from any cause (14). Actually, the incidence of NAH is far more than these due to recall bias, missing detection, and underreporting. Especially for outpatients who manage blood glucose with the target of normal glycemic level, the risk of hypoglycemia will inevitably increase, which is less likely to be recognized and concerned without timely medical guidance. Hence, in view of its universality and perniciousness, predicting the episodes of NAH to minimize hypoglycemic events is significantly meaningful for better diabetes management.

Continuous glucose monitoring (CGM), which provides maximal information about glucose fluctuation levels throughout the day (15), provides an improved opportunity to capture NAH events. CMG has been proved to be superior to daily self-monitoring blood glucose (SMBG) in the detection of hypoglycemia. In hospitalized patients with type 2 diabetes, the detection rates of hypoglycemia by CMG ranges from 1.6-fold (16), 2.5-fold (17), 4-fold (18) than those by point-of-care capillary glucose testing (POC). Additionally, compared with SMBG, significantly higher percentages of hypoglycemic episodes [(3.8% vs 1.7%) (19); (4.35% vs 1.5%) (20); (90.4% vs 38.5%) (21); (52 vs. 3 events/patient-year) (22)] were detected by CGM, particularly in terms of asymptomatic and nocturnal hypoglycemia (19, 21, 22). Nonetheless, probably due to high cost and technical complexity (23), CGM is still underutilized in the real world. SMBG, on the other hand, remains the basic approach for glycemic management in daily life, which is widely used because of its familiarity, convenience and relatively low cost for long-term daily diabetes management (23). We propose that by combining the advantages of CGM and SMBG, that is, based on precisely capturing hypoglycemia by CGM, predicting the episodes of hypoglycemia through glycemic indicators monitored by SMBG may be possible to prevent hypoglycemia with accuracy and convenience.

Glycemic variability, characterized by extreme glucose excursions, is associated with the risk of overall symptomatic, nocturnal symptomatic and severe hypoglycemia in patients with diabetes (24), and different indices of glycemic fluctuations have been used to predict hypoglycemia (25–27). However, most of these predictors are obtained by CGM data, and the majority of subjects are hospitalized patients. Relatively few studies focus on outpatients, and efforts are needed to provide easily acquired indictors for outpatients to warn and prevent NAH. In this study, we used CGM device to continuously monitor blood glucose in outpatients with type 2 diabetes to access the episodes of NAH and glycemic variability indices, including glucose standard deviation (SD), mean amplitude of plasma glucose excursions (MAGE) and mean blood glucose (MBG). Simultaneously, the largest amplitude of glycemic excursion (LAGE) was acquired by SMBG without changing patients’ lifestyle and medications. We aimed to clarify the associations of glucose fluctuations and NAH of outpatients with type 2 diabetes, and to explore whether LAGE could independently predict the episodes of NAH, providing a relatively convenient warning index for daily NAH prevention.

## Methods

### Participants

In this study, 313 out-patients with type 2 diabetes who were admitted to the First Affiliated Hospital of Xiamen University from January 2018 to June 2021 were included. All participants wore CGM device at the outpatient clinic, during which medication use was not affected. Pregnant and perioperative patients were excluded. Body mass index (BMI) was calculated as the weight in kilograms divided by the square of height in meters. HbA1c and C-peptide were detected by the Laboratory Department of the First Affiliated Hospital of Xiamen University. This study was approved by the ethics committees of the First Affiliated Hospital of Xiamen University. Written informed consent was obtained from all subjects.

### Continuous Glucose Monitoring

A iPro™2 CGM system (Medtronic, Minimed, Inc. Northridge, CA), which is extensively used in detecting low glucose levels with validated reproducibility and reliability (28), was used in this study to monitor glucose fluctuations. After wearing the CGM device, participants returned home and resumed normal activities for consecutive 72 hours. NAH was defined as hypoglycemia (<3.9mmol/L) occurring between 0 am and 6 am. We obtained glycemic variability indices from CGM, including glucose SD, MAGE, MBG, time in range (TIR; 3.9-10.0 mmol/L), time below range (TBR; <3.9 mmol/L).

### Self-Monitoring Blood Glucose

The OneTouch UltraVue^®^ (Johnson and Johnson K.K., Tokyo, Japan) device and ACCU-CHEK Performa (Roche, Switzerland) glucometer were used for SMBG. Each subject used the same glucometer during the 72-h study period. Participants were guided to conduct blood glucose self-monitoring four times daily - prior to meals and bedtime. The maximum range of daily blood glucose fluctuation was obtained by subtracting the minimum from the maximum value, and then the daily maximum ranges of the 72 hours were equilibrated to obtain the LAGE value.

### Statistical Analyses

Data were expressed as mean ± standard deviation (SD) for normally distributed variables, median (25th percentile, 75th percentile) for non-normally distributed variables, and percentages for categorical variables. The significance of differences between the two groups was assessed using t test or Kruskal-Wallis test for quantitative data, and Chi-square test for categorical data. The associations between LAGE and the incidence of NAH and TBR were analyzed by a logistic regression model and a linear regression model, respectively. The interaction of LAGE and potential risk factors of NAH was performed by logistic regression analysis as well. We run receiver operating characteristic (ROC) curve analysis to demonstrate the sensitivity, specificity and optimal cut-off value of LAGE for predicting NAH. The predictive validity was quantified as areas under the ROC curve. A P<0.05 was considered to be statistically significant. All statistical analyses were conducted with SAS version 9.3.

## Results

### Clinical Characteristics and Blood Glucose Monitoring Results

**Table 1** shows the characteristics of total 313 outpatients with type 2 diabetes, including 76 patients with NAH and 237 patients without. There were no significant differences in age, duration of diabetes, BMI, HbA1c, concentration of C-peptide, and drug uses (metformin, dipeptidyl peptidase-4 inhibitor, alpha-glucosidase inhibitors, thiazolidinedione, glucagon-like peptide-1 receptor agonists, sulfonylurea, sodium–glucose cotransporter 2 inhibitors, long-acting insulin, premixed insulin, short-acting insulin) between the two groups. Further, we found that diabetic comorbidities (hypertension, hyperlipidemia, fatty liver, cardio-cerebral vascular disease) and diabetic complications (diabetic retinopathy, diabetic peripheral neuropathy, diabetic peripheral vascular disease, diabetic nephropathy, diabetic foot) were not statistically different. The glycemic variability indices, including glucose SD (2.4 ± 0.9 mmol/L vs 1.7 ± 0.9 mmol/L, p<0.001), MAGE (5.2 ± 2.1 mmol/L vs 3.7 ± 2.0 mmol/L, p <0.001) and LAGE (4.6 ± 2.3 mmol/L vs 3.8 ± 1.9 mmol/L, p=0.007), were higher in patients with NAH than those in patients without, while MBG (7.5 ± 1.5 mmol/L vs 8.4 ± 2.2 mmol/L, p=0.002) was lower. TIR [0.8 (0.7,0.9) vs 0.9 (0.7,1.0)] did not differ statistically in subjects with or without NAH.

**Table 1:** Clinical characteristics and blood glucose monitoring results.

	Without NAH	With NAH	P value
	(n = 237)	(n = 76)	
Age (years)	55.4 ± 14.1	51.7 ± 15.5	0.058
Duration of diabetes (years)	6.0 (2.0,11.0)	5.0 (3.0,13.0)	0.445
BMI (kg/m^2^)	23.2 ± 3.1	22.6 ± 3.2	0.217
HbA1c (%)	7.0 ± 1.5	7.0 ± 1.5	0.933
C-peptide (ng/mL)	1.5 (0.9,2.3)	0.9 (0.3,1.9)	5.691
Medication (%)			
Metformin	64 (28.2)	21 (29.2)	0.873
DPP-4i	34 (15)	11 (15.3)	0.951
α-GI	46 (20.3)	11 (15.3)	0.348
TZD	5 (2.2)	3 (4.2)	0.368
GLP-1RA	4 (1.8)	1 (1.4)	0.830
SU	49 (21.6)	18 (25)	0.545
SGLT-2i	8 (3.5)	2 (2.8)	0.759
Long-acting insulin	50 (22.0)	15 (20.8)	0.831
Premixed insulin	27 (11.9)	6 (8.3)	0.401
Short-acting insulin	38 (16.7)	11 (15.3)	0.770
Comorbidity (%)			
Hypertension	28 (12.3)	9 (12.5)	0.970
Hyperlipidemia	35 (15.4)	12 (16.7)	0.800
Fatty liver	11 (4.9)	5 (6.9)	0.491
CCVD	19 (8.4)	7 (9.7)	0.723
Complication (%)			
DR	31 (13.7)	13 (18.1)	0.359
DPN	31 (13.7)	9 (12.5)	0.802
DPVD	10 (4.4)	4 (18.1)	0.687
DN	11 (4.9)	4 (5.6)	0.810
DF	2 (0.9)	0 (0)	0.424
CGM data			
SD (mmol/L)	1.7 ± 0.9	2.4 ± 0.9	<0.001
MBG (mmol/L)	8.4 ± 2.2	7.5 ± 1.5	0.002
MAGE (mmol/L)	3.7 ± 2.0	5.2 ± 2.1	<0.001
TIR (%)	90 (70,100)	80 (70,90)	9.895
SMBG data			
LAGE (mmol/L)	3.8 ± 1.9	4.6 ± 2.3	0.007

NAH, nocturnal asymptomatic hypoglycemia; BMI, body mass index; HbA1c, glycated hemoglobin; DPP-4i, dipeptidyl peptidase-4 inhibitors; α-GI, alpha-glucosidase inhibitors; TZD, thiazolidinedione; GLP-1RA, glucagon-like peptide-1 receptor agonists; SU, sulfonylurea; SGLT-2i, sodium–glucose cotransporter 2 inhibitors; CCVD, cardio-cerebral vascular disease; DR, diabetic retinopathy; DPN, diabetic peripheral neuropathy; DPVD, diabetic peripheral vascular disease; DN, diabetic nephropathy; DF, diabetic foot; CGM, continuous glucose monitoring; SD, glucose standard deviation; MBG, mean blood glucose; MAGE, mean amplitude of plasma glucose excursion; TIR, time in range (3.9–10.0 mmol/L); SMBG, self-monitoring blood glucose; LAGE, largest amplitude of glycemic excursion. P < 0.05 was considered significant.

### Association Between LAGE and the Incidence of NAH and TBR

In **Table 2**, the associations of LAGE with the incidence of NAH and TBR were elucidated by a logistic regression model and a linear regression analysis, respectively. TBR is a key metric for evaluating the degree and severity of hypoglycemia (29), which is more relevant for capturing hypoglycemic events and quantifying their magnitude and duration (30). In model 1 with adjustment for age, BMI, sex, work, hyperlipidemia, complication and medication, LAGE was significantly associated with the increased risk of NAH, with the incidence of NAH [1.189 (1.027-1.378), p=0.021] and TBR [0.008 (0.002-0.014), p=0.013]. In model 2 after adjusting for diabetes duration based on model 1, the same significant result was seen, with the incidence of NAH [1.177 (1.013-1.367), p=0.033] and TBR [0.008 (0.002-0.014), p=0.012]. In model 3 with further adjustment for HbA1c based on model 2, the incidence of NAH increased 1.244-fold (95% CI: 1.057-1.464, p=0.009) and TBR increased 0.009 (95% CI: 0.002-0.016, p=0.007) for 1 mmol/L increase of LAGE.

**Table 2:** Associations between LAGE and the incidence of NAH and TBR.

	The incidence of nocturnal asymptomatic hypoglycemia		TBR	
	OR (95% CI)	P value	Estimateβ (95% CI)	P value
Model 1	1.189 (1.027-1.378)	0.021	0.008 (0.002-0.014)	0.013
Model 2	1.177 (1.013-1.367)	0.033	0.008 (0.002-0.014)	0.012
Model 3	1.244 (1.057-1.464)	0.009	0.009 (0.002-0.016)	0.007

TBR, time below range (<3.9 mmol/L); CI, confidence interval. P < 0.05 was considered significant. Model 1 was adjusted for age, BMI, sex, work, hyperlipidemia, complication and medication. Model 2 was further adjusted for diabetes duration based on model 1. Model 3 was further adjusted for HbA1c based on model 2.

### Association Between LAGE and Potential Risk Factors on the Risk of NAH

In order to further determine whether LAGE was independently correlated with NAH, the logistic regression model was further performed according to potential risk factors. **Table 3** shows that no significant interactions were found between LAGE and sex (p for interaction= 0.732), age (p for interaction= 0.187), HbA1c (p for interaction= 0.877), duration of diabetes (p for interaction= 0.734), BMI (p for interaction= 0.864) and insulin therapy (p for interaction= 0.474) on the risk of NAH.

**Table 3:** Association between LAGE and potential risk factors on the risk of NAH.

		With nocturnal asymptomatic hypoglycemia		
	Total	OR (95% CI)	P value	Interaction
Sex				0.732
Men	175	1.36 (1.04-1.77)	0.023	
Women	138	1.19 (0.95-1.48)	0.130	
Age(years)				0.187
<55	163	1.41 (1.09-1.82)	0.009	
≥55	150	1.13 (0.88-1.46)	0.357	
HbA1c (%)				0.877
≤7	217	1.35 (1.04-1.74)	0.025	
>7	96	1.27 (0.99-1.62)	0.059	
Duration of diabetes (years)				0.734
≤5	164	1.20 (0.90-1.61)	0.218	
>5	149	1.32 (1.06-1.64)	0.014	
BMI (kg/m^2)^				0.864
<24	216	1.09 (0.86-1.39)	0.471	
≥24	97	1.38 (1.04-1.83)	0.024	
Insulin therapy				
With	96	1.39 (1.07-1.81)	0.123	0.474
Without	217	1.13 (0.89-1.44)	0.313	

### ROC Curve of LAGE for the Prediction of NAH

The ideal cutoff value of LAGE for the prediction of NAH was 3.48 mmol/L with 66.7% sensitivity and 50% specificity. The area under the ROC curve (AUC) was 0.587 (95%CI: 0.509-0.665).

## Discussion

In this study, we clarified that higher levels of glucose SD, MAGE and LAGE and lower levels of MBG were strongly associated with NAH in outpatients with type 2 diabetes, and demonstrated that LAGE may be an independent predictor of NAH, irrespective of HbA1c level and other potential risk factors.

It has been shown that frequent hypoglycemia often occurred with a greater level of glucose fluctuations (31). In addition, glycemic variability has been suggested to be a potential indicator of diabetes complications (32) and severe hypoglycemia (25). Through re-analyzing the Diabetes Control and Complications Trial (DCCT) data, Kilpatrick et al. found that MBG and glycemic variability each have an independent role in increased risk of hypoglycemia in type 1 diabetes. The incidence of time to first hypoglycemic event increased 1.05-fold for each 1 mmol/l decrease in MBG and 1.07-fold for every 1 mmol/l increase in glucose SD. After adjusting for HbA1c, a 1 mmol/l increase in SD was associated with a 1.09-fold increased risk of a first event (26). Saisho et al. reported that glucose SD and other glycemic variability indices were more strongly correlated with hypoglycemia compared with MBG, and the combination of MBG and glucose SD was useful for predicting hypoglycemia in diabetes patients (33). Service et al. suggested that a high MAGE was a vital characteristic of glucose instability, which was more accurate than other indexes of glycemic fluctuation (34). Another study of 5-day consecutive CGM showed that hypoglycemic patients had lower MBG and higher glucose SD compared to non-hypoglycemic patients, with no statistical difference of HbA1c (35), which is consistent with our research. In this study, patients with NAH manifested as higher levels of SD, MAGE and LAGE and lower level of MBG. After adjusting for possible interference factors, there were still significant associations of LAGE with NAH and TBR. Moreover, no significant interactions were observed between LAGE and potential risk factors, indicating that LAGE could be an independent predictor of NAH in patients with type 2 diabetes. According to the CGM data conducted by Zhou el al. in Shanghai, China, MAGE <3.9 mmol/L and SD <1.4 mmol/L were recommended as the normal reference ranges for glycemic variability in Chinese adults (36), and LAGE <5.7mmol/L was recommended in normal glucose tolerance people (37). To the best of our knowledge, the value of LAGE in determining the risk of NAH has not been reported. Here, we showed that LAGE greater than 3.48 mmol/L could act as a warning alarm for high risk of NAH in outpatients with type 2 diabetes.

Although the diabetes management has focused on HbA1c, as an index reflecting recent average blood glucose levels, HbA1c could not accurately portray the frequency of hypoglycemia and glucose fluctuations (35). HbA1c was reported to minimally contributes to hypoglycemia risk in type 2 diabetes and has no relation to hypoglycemia in type 1 diabetes, while the variability in glucose levels showed great promise as better predictors (38).In this study, HbA1c did not differ between the two groups, which was consistent with other researches (28, 35), confirming the ability of LAGE beyond HbA1c for predicting NAH.

Our research has the following strengths. Firstly, the subjects in this study are outpatients with type 2 diabetes, the diabetic condition of whom are generally considered to be in stable, so NAH is not seriously concerned among these individuals. In addition, as they usually aim for normal blood glucose level, NAH is more likely to occur and leads to serious complications without timely medical assistances. Therefore, the prediction and prevention of NAH is extremely meaningful in such a population. Secondly, in spite of some advantages of CGM, long-term wearing of CGM devices for outpatients is currently impractical. Nevertheless, LAGE based on SMBG is easily calculated and convenient to make a rapid assessment for NAH risk. Thirdly, all data were acquired without changing patients’ lifestyle and medications, which reflects the true daily glucose fluctuations, improving the reliability of LAGE as an independent predictor of NAH.

Several limitations of this study should be noted. Firstly, the sample size included in this study is relatively small. A study with a larger sample size and longer duration is needed to further consolidate the results of this study. Secondly, subjects included in this study are patients with type 2 diabetes, thus our findings may not be applicable to all diabetes patients, especially patients with type 1 diabetes. Thirdly, other factors that may affect glycemic variability, such as exercise, food intake and beta-cell function, were not investigated in the current study. In the near future, we will continue to study with a larger sample size and try to combine these factors for analysis to improve the specificity and sensitivity of the prediction of NAH. Finally, severe hypoglycemia (<3.0 mmol/L) is also critical. Because there were only 30 patients with severe hypoglycemia in this study, which may affect statistical power, there was no statistical difference between LAGE and severe hypoglycemia (data not shown). Our subsequent studies will also include more patients with blood glucose less than 3.0 mmol/L to clarify the association between LAGE and severe hypoglycemia.

## Conclusions

In conclusion, our study showed that higher glycemic variability is strongly associated with higher risk of NAH, and proposed LAGE could be an independent predictor of NAH for outpatients with type 2 diabetes. LAGE greater than 3.48 mmol/L could act as a warning alarm for high risk of NAH. Taking the convenience and feasibility of SBMG into account, a real-time alarm based on LAGE may minimize NAH exposure to further achieve better diabetes management. We hope our research can serve as a reference that helps in hypoglycemia prevention.

## Data Availability Statement

The datasets generated during the current study are available from the corresponding author on reasonable request.

## Ethics Statement

The studies involving human participants were reviewed and approved by The First Affiliated Hospital of Xiamen University. The patients/participants provided their written informed consent to participate in this study.

## Author Contributions

SW researched the data and wrote the manuscript. ZT researched the data and edited the manuscript. TW and QS integrated the data. PH, LW, and WL contributed to the discussion. HS and ML contributed to the introduction. XS analyzed the data. XL reviewed and edited the manuscript. All authors approved the manuscript.

## Funding

This work was supported by the Natural Science Foundation of Fujian Province, China (No.2021J011363).

## Conflict of Interest

The authors declare that the research was conducted in the absence of any commercial or financial relationships that could be construed as a potential conflict of interest.

## Publisher’s Note

All claims expressed in this article are solely those of the authors and do not necessarily represent those of their affiliated organizations, or those of the publisher, the editors and the reviewers. Any product that may be evaluated in this article, or claim that may be made by its manufacturer, is not guaranteed or endorsed by the publisher.

## References

[1] Abraham MB Jones TW Naranjo D Karges B Oduwole A Tauschmann M. Ispad Clinical Practice Consensus Guidelines 2018: Assessment and Management of Hypoglycemia in Children and Adolescents With Diabetes. Pediatr Diabetes (2018) 19 Suppl 27:178–92. doi: 10.1111/pedi.12698 29869358

[2] Group AC Patel A MacMahon S Chalmers J Neal B Billot L. Intensive Blood Glucose Control and Vascular Outcomes in Patients With Type 2 Diabetes. N Engl J Med (2008) 358(24):2560–72. doi: 10.1056/NEJMoa0802987 18539916

[3] Duckworth W Abraira C Moritz T Reda D Emanuele N Reaven PD. Glucose Control and Vascular Complications in Veterans With Type 2 Diabetes. N Engl J Med (2009) 360(2):129–39. doi: 10.1056/NEJMoa0808431 19092145

[4] Pettersson B Rosenqvist U Deleskog A Journath G Wandell P. Self-Reported Experience of Hypoglycemia Among Adults With Type 2 Diabetes Mellitus (Exhype). Diabetes Res Clin Pract (2011) 92(1):19–25. doi: 10.1016/j.diabres.2010.12.005 21195501

[5] Stargardt T Gonder-Frederick L Krobot KJ Alexander CM. Fear of Hypoglycaemia: Defining a Minimum Clinically Important Difference in Patients With Type 2 Diabetes. Health Qual Life Outcomes (2009) 7:91. doi: 10.1186/1477-7525-7-91 19849828 PMC2770562

[6] Chan SP Ji LN Nitiyanant W Baik SH Sheu WH. Hypoglycemic Symptoms in Patients With Type 2 Diabetes in Asia-Pacific-Real-Life Effectiveness and Care Patterns of Diabetes Management: The Recap-Dm Study. Diabetes Res Clin Pract (2010) 89(2):e30–2. doi: 10.1016/j.diabres.2010.05.008 20541826

[7] Marrett E Radican L Davies MJ Zhang Q. Assessment of Severity and Frequency of Self-Reported Hypoglycemia on Quality of Life in Patients With Type 2 Diabetes Treated With Oral Antihyperglycemic Agents: A Survey Study. BMC Res Notes (2011) 4:251. doi: 10.1186/1756-0500-4-251 21777428 PMC3148563

[8] McCoy RG Van Houten HK Ziegenfuss JY Shah ND Wermers RA Smith SA. Self-Report of Hypoglycemia and Health-Related Quality of Life in Patients With Type 1 and Type 2 Diabetes. Endocr Pract (2013) 19(5):792–9. doi: 10.4158/EP12382.OR 23757608

[9] Patterson CC Dahlquist G Harjutsalo V Joner G Feltbower RG Svensson J. Early Mortality in Eurodiab Population-Based Cohorts of Type 1 Diabetes Diagnosed in Childhood Since 1989. Diabetologia (2007) 50(12):2439–42. doi: 10.1007/s00125-007-0824-8 17901942

[10] Feltbower RG Bodansky HJ Patterson CC Parslow RC Stephenson CR Reynolds C. Acute Complications and Drug Misuse Are Important Causes of Death for Children and Young Adults With Type 1 Diabetes: Results From the Yorkshire Register of Diabetes in Children and Young Adults. Diabetes Care (2008) 31(5):922–6. doi: 10.2337/dc07-2029 18285550

[11] Skrivarhaug T Bangstad HJ Stene LC Sandvik L Hanssen KF Joner G. Long-Term Mortality in a Nationwide Cohort of Childhood-Onset Type 1 Diabetic Patients in Norway. Diabetologia (2006) 49(2):298–305. doi: 10.1007/s00125-005-0082-6 16365724

[12] Edelman SV Blose JS. The Impact of Nocturnal Hypoglycemia on Clinical and Cost-Related Issues in Patients With Type 1 and Type 2 Diabetes. Diabetes Educ (2014) 40(3):269–79. doi: 10.1177/0145721714529608 24695260

[13] Lamounier RN Geloneze B Leite SO Montenegro R Jr. Zajdenverg L Fernandes M. Hypoglycemia Incidence and Awareness Among Insulin-Treated Patients With Diabetes: The Hat Study in Brazil. Diabetol Metab Syndr (2018) 10:83. doi: 10.1186/s13098-018-0379-5 30479669 PMC6249957

[14] International Hypoglycaemia Study G. Hypoglycaemia, Cardiovascular Disease, and Mortality in Diabetes: Epidemiology, Pathogenesis, and Management. Lancet Diabetes Endocrinol (2019) 7(5):385–96. doi: 10.1016/S2213-8587(18)30315-2 30926258

[15] Klonoff DC. Continuous Glucose Monitoring: Roadmap for 21st Century Diabetes Therapy. Diabetes Care (2005) 28(5):1231–9. doi: 10.2337/diacare.28.5.1231 15855600

[16] Levitt DL Spanakis EK Ryan KA Silver KD. Insulin Pump and Continuous Glucose Monitor Initiation in Hospitalized Patients With Type 2 Diabetes Mellitus. Diabetes Technol Ther (2018) 20(1):32–8. doi: 10.1089/dia.2017.0250 PMC5770096 29293367

[17] Gomez AM Umpierrez GE Munoz OM Herrera F Rubio C Aschner P. Continuous Glucose Monitoring Versus Capillary Point-Of-Care Testing for Inpatient Glycemic Control in Type 2 Diabetes Patients Hospitalized in the General Ward and Treated With a Basal Bolus Insulin Regimen. J Diabetes Sci Technol (2015) 10(2):325–9. doi: 10.1177/1932296815602905 PMC4773955 26330394

[18] Galindo RJ Migdal AL Davis GM Urrutia MA Albury B Zambrano C. Comparison of the Freestyle Libre Pro Flash Continuous Glucose Monitoring (Cgm) System and Point-Of-Care Capillary Glucose Testing in Hospitalized Patients With Type 2 Diabetes Treated With Basal-Bolus Insulin Regimen. Diabetes Care (2020) 43(11):2730–5. doi: 10.2337/dc19-2073 PMC7809713 32641372

[19] Pazos-Couselo M Garcia-Lopez JM Gonzalez-Rodriguez M Gude F Mayan-Santos JM Rodriguez-Segade S. High Incidence of Hypoglycemia in Stable Insulin-Treated Type 2 Diabetes Mellitus: Continuous Glucose Monitoring Vs. Self-Monitored Blood Glucose. Observational Prospective Study. Can J Diabetes (2015) 39(5):428–33. doi: 10.1016/j.jcjd.2015.05.007 26254702

[20] Afandi B Hassanein M Roubi S Nagelkerke N. The Value of Continuous Glucose Monitoring and Self-Monitoring of Blood Glucose in Patients With Gestational Diabetes Mellitus During Ramadan Fasting. Diabetes Res Clin Pract (2019) 151:260–4. doi: 10.1016/j.diabres.2019.01.036 30822494

[21] Shivaprasad C Gautham K Shah K Gupta S Palani P Anupam B. Continuous Glucose Monitoring for the Detection of Hypoglycemia in Patients With Diabetes of the Exocrine Pancreas. J Diabetes Sci Technol (2021) 15(6):1313–9. doi: 10.1177/1932296820974748 PMC8655303 33322930

[22] Agesen RM Kristensen PL Beck-Nielsen H Norgaard K Perrild H Jensen T. Effect of Insulin Analogs on Frequency of Non-Severe Hypoglycemia in Patients With Type 1 Diabetes Prone to Severe Hypoglycemia: Much Higher Rates Detected by Continuous Glucose Monitoring Than by Self-Monitoring of Blood Glucose-The Hypoana Trial. Diabetes Technol Ther (2018) 20(3):247–56. doi: 10.1089/dia.2017.0372 29565719

[23] Ajjan RA. How Can We Realize the Clinical Benefits of Continuous Glucose Monitoring? Diabetes Technol Ther (2017) 19(S2):S27–36. doi: 10.1089/dia.2017.0021 PMC5444484 28541132

[24] DeVries JH Bailey TS Bhargava A Gerety G Gumprecht J Heller S. Day-To-Day Fasting Self-Monitored Blood Glucose Variability Is Associated With Risk of Hypoglycaemia in Insulin-Treated Patients With Type 1 and Type 2 Diabetes: A *Post Hoc* Analysis of the Switch Trials. Diabetes Obes Metab (2019) 21(3):622–30. doi: 10.1111/dom.13565 PMC6587774 30362250

[25] Siegelaar SE Holleman F Hoekstra JB DeVries JH. Glucose Variability; Does It Matter? Endocr Rev (2010) 31(2):171–82. doi: 10.1210/er.2009-0021 19966012

[26] Kilpatrick ES Rigby AS Goode K Atkin SL. Relating Mean Blood Glucose and Glucose Variability to the Risk of Multiple Episodes of Hypoglycaemia in Type 1 Diabetes. Diabetologia (2007) 50(12):2553–61. doi: 10.1007/s00125-007-0820-z 17882397

[27] Klimontov VV Myakina NE. Glucose Variability Indices Predict the Episodes of Nocturnal Hypoglycemia in Elderly Type 2 Diabetic Patients Treated With Insulin. Diabetes Metab Syndr (2017) 11(2):119–24. doi: 10.1016/j.dsx.2016.08.023 27569727

[28] Woodward A Weston P Casson IF Gill GV. Nocturnal Hypoglycaemia in Type 1 Diabetes–Frequency and Predictive Factors. QJM (2009) 102(9):603–7. doi: 10.1093/qjmed/hcp082 19574471

[29] Advani A. Positioning Time in Range in Diabetes Management. Diabetologia (2020) 63(2):242–52. doi: 10.1007/s00125-019-05027-0 31701199

[30] Monnier L Colette C Owens D. Application of Medium-Term Metrics for Assessing Glucose Homoeostasis: Usefulness, Strengths and Weaknesses. Diabetes Metab (2021) 47(2):101173. doi: 10.1016/j.diabet.2020.06.004 32561428

[31] He H Wang C Chen DW Xiao J Yang XJ Lu LF. Characteristics of 72 H Glucose Profiles Detected by Continuous Glucose Monitoring System in Patients With Insulinoma. Sichuan Da Xue Xue Bao Yi Xue Ban (2014) 45(4):623–7. 25286688

[32] Nalysnyk L Hernandez-Medina M Krishnarajah G. Glycaemic Variability and Complications in Patients With Diabetes Mellitus: Evidence From a Systematic Review of the Literature. Diabetes Obes Metab (2010) 12(4):288–98. doi: 10.1111/j.1463-1326.2009.01160.x 20380649

[33] Saisho Y Tanaka C Tanaka K Roberts R Abe T Tanaka M. Relationships Among Different Glycemic Variability Indices Obtained by Continuous Glucose Monitoring. Prim Care Diabetes (2015) 9(4):290–6. doi: 10.1016/j.pcd.2014.10.001 25456706

[34] Service FJ O'Brien PC Rizza RA. Measurements of Glucose Control. Diabetes Care (1987) 10(2):225–37. doi: 10.2337/diacare.10.2.225 3582083

[35] Uemura F Okada Y Torimoto K Tanaka Y. Relation Between Hypoglycemia and Glycemic Variability in Type 2 Diabetes Patients With Insulin Therapy: A Study Based on Continuous Glucose Monitoring. Diabetes Technol Ther (2018) 20(2):140–6. doi: 10.1089/dia.2017.0306 29293363

[36] Zhou J Li H Ran X Yang W Li Q Peng Y. Establishment of Normal Reference Ranges for Glycemic Variability in Chinese Subjects Using Continuous Glucose Monitoring. Med Sci Monit (2011) 17(1):CR9–13. doi: 10.12659/msm.881318 21169911 PMC3524682

[37] Zhou J Jia WP Yu M Yu HY Bao YQ Ma XJ. The Reference Values of Glycemic Parameters for Continuous Glucose Monitoring and Its Clinical Application. Zhonghua Nei Ke Za Zhi (2007) 46(3):189–92. 17547797

[38] Rama Chandran S Tay WL Lye WK Lim LL Ratnasingam J Tan ATB. Beyond Hba1c: Comparing Glycemic Variability and Glycemic Indices in Predicting Hypoglycemia in Type 1 and Type 2 Diabetes. Diabetes Technol Ther (2018) 20(5):353–62. doi: 10.1089/dia.2017.0388 29688755

